# On harm thresholds and living organ donation: must the living donor benefit, on balance, from his donation?

**DOI:** 10.1007/s11019-017-9778-x

**Published:** 2017-05-19

**Authors:** Nicola Jane Williams

**Affiliations:** Department of Politics, Philosophy and Religion, County South, Lancaster University, Bailrigg, Lancaster, Lancashire LA1 4YW UK

**Keywords:** Living organ donation, Transplantation ethics, Autonomy, Beneficence, Harm

## Abstract

For the majority of scholars concerned with the ethics of living organ donation, inflicting moderate harms on competent volunteers in order to save the lives or increase the life chances of others is held to be justifiable provided certain conditions are met. These conditions tend to include one, or more commonly, some combination of the following: (1) The living donor provides valid consent to donation. (2) Living donation produces an overall positive balance of harm–benefit for donors and recipients which cannot be obtained in a less harmful manner. (3) Donation is not liable to cause significant and long-term morbidity to, or the death of, the donor. This paper critically examines the suggestion that these criteria are not sufficient to offer a general account of justified living organ donation in the context of competent volunteers and that key to justified living organ donation is that donors receive sufficient benefits from their donation that these outweigh the harms they suffer. However, although this view—termed here ‘The Donor Benefit Standard’—directs welcome attention to the many and complex motives which may underlie living organ donation, this paper ultimately concludes that given the threats this position poses to individual autonomy and the lives of those in need of organ transplants ‘The Donor Benefit Standard’ should ultimately be rejected.

## Introduction

The living donation of organs has been permitted since the dawn of the transplantation era when organ retrieval techniques, tissue preservation procedures, and immunosuppressive medications were not sufficiently developed to allow for the safe transplantation of organs from deceased donors. At this time, in the absence of retrieval from living donors, those in need of organ transplants would almost certainly die. Thus, transplant surgeons viewed living donation as a temporary and uncomfortable necessity, expressing the hope that in the future the need to resort to living donors would be made obsolete as “organs obtained solely from cadavers could be used with a high expectation of survival” (Starzl 1967, pp. 35–6). Yet, although deceased donor transplantation has been possible for decades, living organ donation continues, contrary to the hopes of the first transplant physicians, apace.

Indeed, the practice is now so commonplace that: in the UK 34% (NHS 2015, p. 38) and worldwide 46% (World Health Organisation 2013) of kidneys for transplantation are so obtained; legal and professional guidelines and regulations regarding suitable living donors have been relaxed such that donors who would not have been considered even 15 years ago—for reasons of poor tissue matching, medical contraindications or questionable motivation—are now being used; and organ retrieval from living donors has been extended from cases of vital and semi-vital organ and composite tissue transplants to quality of life transplants such as uterine (Brannstrom et al. 2014) and ovary (Silber et al. 2005) transplantation.

Yet, despite how widespread this practice has become there remains a general sense of unease amongst many medical professionals and other interested parties regarding the performance of harmful/risky and medically unnecessary surgery on one for the sake of saving or improving the life of another. This is not necessarily morally problematic. For, although no transplant surgeon would welcome the prospect of causing significant harm to living donors, living organ donation is now generally justified by appeals to respect for the autonomy of candidate donors; the ever-widening gap between those in need of donor organs and the number of deceased donor organs available; and the superior longevity and quality associated with organs obtained from the living. Thus, it is generally held that until such time that living donation neither bridges the transplant gap nor offers significant benefits to recipients over deceased donation or in vitro and artificial organs, this practice may be justified provided certain conditions are met.

These conditions tend to include, for competent donors, the following requirements:

1. The living donor provides his valid consent.
2. Living donation produces an overall positive balance of harm–benefit for donors and recipients which cannot be obtained in a less harmful manner.
3. Donation is not liable to cause significant and long-term morbidity to, or the death of the donor.

For some authors, however, that an instance of living donation fulfils these three criteria is not deemed sufficient or necessary to offer a general account of justified living organ donation. In terms of necessity, for example, a strict libertarian may hold that valid consent is the only requirement and a utilitarian may hold that to prove justifiable an instance of living donation must produce only more benefit than harm. For the purposes of this paper however, questions of necessity regarding these three criteria are sidelined and it is assumed that all three of the requirements for justified living organ donation listed above should be deemed necessary for justified living organ donation. Thus, the focus here is on the question of sufficiency: of whether, in order to offer a general account of justified living organ donation, these three criteria must be amended or supplemented by additional criteria such that *more stringent restrictions are imposed* on the circumstances in which living donors may permissibly be used.

Given the volume of literature concerning the ethics of living organ transplantation it is perhaps unsurprising that there exist several candidates for additional criteria or stronger versions of these three criteria. However, this paper is concerned with just one: a position advanced by Aaron Spital in numerous papers which holds that a *physician is only justified in removing organs from the living where she is convinced that the donor will benefit, on balance, from their donation* (See: Spital 2004a, b, 2005a, b, 2006; Spital and Jacobs 2007; Spital and Taylor 2007, 2008). Thus, Spital suggests that the third criterion, understood here as imposing a threshold on the extent to which a living donor may justifiably be harmed as a result of their donation, should take a far stronger form, requiring not just that donation be unlikely to cause grave harms to, or the death of the donor, but that:

3*. Donation is reasonably expected to provide benefits for the donor that outweigh the harms she may suffer.

This paper explores and examines this suggestion. It thus begins with an exploration of the claim that a threshold should be placed on the extent to which a living organ donor may be harmed as a result of his donation, and the possible justifications that may underlie such a policy. The question of whether what will be referred to as ‘the donor benefit standard’ for justified living organ donation should be preferred to more permissive versions of the donor harm threshold is then discussed. Ultimately however, it is concluded that—given several shortcomings associated with this position, and the fact that any policy which imposes further limits on the number of organs made available for transplantation is one with grave implications for organ recipients and thus requires *strong* justification—donor benefit is not a requirement of justified living organ donation.

## The donor harm threshold

Although considerations of harm–benefit and valid consent have always received, and continue to receive, significant attention in the ethics and policy literature regarding living donation, comparatively little philosophical attention has been paid to the suggestion that limits should be placed on the extent to which living organ donors may be harmed as a result of their donation. Yet, despite this, few but the most committed of libertarian and utilitarian scholars, are likely to condone *in either principle or practice* the retrieval of organs such as hearts, second kidneys, and other whole vital organs from the living. Thus, it is generally held that just as an instance of living organ donation will be permissible (in the case of competent donors) only where organs are freely given and where donation is expected (and necessary) to produce, an all-things-considered benefit for those holding a stake in the donation, a threshold should be placed on the extent to which living organ donors may be harmed as a result of their donation. Where this threshold should lie is a matter of debate but it is generally held that it requires, *at the very least*, that donation not be certain, or highly likely, to cause death.

Yet, why should a threshold be placed on the harms that living organ donors are permitted to endure? Why should an altruist not be permitted to forfeit his life and donate his organs and tissues to needy strangers, a husband be proscribed from sacrificing himself in order to donate his heart to his much-loved wife, or a father be forbidden from risking death and accepting a life on dialysis in order to donate a second kidney to his daughter? Unlike requirements of informed consent and the production of a favorable balance of harm to benefit which may be seen straightforwardly to reflect the value of respect for individual autonomy and consequentialist moral reasoning, the imposition of a donor harm threshold may be motivated by a number of different considerations.

The weakest versions of the donor harm threshold may, for example, be justified via appeal to a corollary of the dead donor rule in deceased organ donation. This rule, often underpinned by the view that the deliberate/foreseen killing of innocents is never permissible, requires that “a human being must be dead before life prolonging organs (e.g. the heart, liver, or lungs) can be procured for transplant or other purposes.” (Veatch and Ross 2015, p. 39) Thus, on such an approach, even where an instance of organ donation is both autonomous and liable to create an overall benefit, cases of donation where donor mortality is certain or highly likely will always be deemed impermissible.

For those who do not take such a decisive stance against intentional or foreseen killing however, other considerations may also be proposed as justifying the imposition of both stronger and weaker versions of the donor harm threshold. Some, such as Carl Elliott, have suggested that placing a limit on the extent to which donors may be harmed may well be justified by reference to considerations regarding the ethics both of receiving gifts and benefiting from the sacrifices of others. On this view, although an intended organ donor may not act wrongly by offering his organs to another when to do so is likely to come at great personal cost, the intended recipient/s and indeed the donor physician may act wrongly in accepting such an offer. For, although our society often heaps praise on those who sacrifice their lives or long-term wellbeing for others, it is generally not deemed so admirable to *encourage* self-sacrificial behaviour in order to gain personal benefits. Thus, Elliot notes:

[A]ccepting a sacrifice of great magnitude is not mere passive acquiescence, devoid of any moral import. If I allow someone else to risk his life or health for my own sake, I am endorsing his self-sacrifice and agreeing to profit by it. Now, of course, if the risk to the donor were very small… an offer like this would be difficult to refuse, and accepting it would surely be justified. But what if the risk were very high? What would we think of a person who would take advantage of a donor’s willingness to take life-threatening risks? What would we think of a person who would accept a heart from a living donor? Unless the circumstances were extraordinary, most of us would think very badly of a person who would agree to, and take advantage of, a sacrifice of this magnitude… his would be an act of failure… a lapse of moral nerve (Elliott 1995, pp. 93–94).

Indeed, the imposition of a donor harm threshold may also be motivated by the very considerations that it supplements and limits. For, just as consequentialist moral reasoning—which justifies or condemns actions by reference to the extent to which they produce (or conform to rules which produce) optimal consequences—leads to the requirement that instances of living organ donation should be permitted only where they produce (and are necessary to produce) an overall positive balance of harm to benefit for those holding a stake in the donation, it may also be used to justify a moderate version of the donor harm threshold. After all, although consequentialist moral reasoning can, *in theory*, justify practices such as organ donation suicide and, indeed, the conscription of vital organs from the living—as in the case of John Harris’ survival lottery (1975) – where to do so would produce optimal consequences, this is unlikely to be the case in practice. In reality, allowing these and other extremely risky instances of living organ donation would be unlikely to produce optimal consequences for at least two reasons. First, the presence of a significant risk of donor mortality or severe and long-term morbidity points strongly to the suggestion that the use of an alternative treatment or source of donor organs (either living or deceased) would be produce a more favourable balance of harm to benefit. Second, however, even where alternatives would not likely result in better outcomes for immediate stakeholders, allowing extremely risky forms of living organ donation could result in wider negative consequences. Increases in rates of donor morbidity and mortality could, for example, lead to both the closing down of transplant programmes by administrators concerned to improve hospital reputations regarding such statistics, and a reduction in the number of willing living donors in the future who may be discouraged by inflated statistics regarding donor risk.

Similarly, just as respect for autonomy motivates the requirement that the retrieval of organs from the living should be permitted only where a donor provides his valid consent to donation, so too may it motivate the requirement that harms to donors fall below some acceptable threshold. It may be suggested, for example, that instances of living donation where the harms to donors are both certain and grave *call into question* or, more strongly, *invalidate* donor consent. The strong claim that certain and grave harm may invalidate donor consent is, of course, hard to justify. Even on the most demanding accounts of autonomy, where autonomous action is perceived as synonymous with rational action, few would be willing to hold that acts of extreme self-sacrifice are necessarily irrational. Yet, whilst this is so, it does seem that the presence of significant risks of grave harm may well call the validity of donor consent into question. That is, their presence may act as a ‘red flag’, suggesting that it is prudent to reconsider how clearly information regarding possible risks has been conveyed and the extent to which their consent is truly autonomous. For, whilst many are willing to suffer moderate harm for the sake of advancing the interests/welfare of another (particularly when that other is someone with whom they share close emotional ties) very few are willing to sacrifice their very lives for this purpose.

## The donor benefit standard

[B]ecause living organ donation *always* involves the possibility of serious harm, the donor’s physician should consider this practice justified only when it is anticipated that there will be *benefits for the donor that exceed the risks to the donor*; benefit for the recipient alone won’t do. (Spital 2004a, p. 105)

As can be seen in the preceding section several arguments may be advanced in favour of imposing a threshold on the extent to which a living donor may be harmed as a result of his donation. However, while such arguments may justify the refusal of living heart donors and offer good reason to bar living donation in cases where donation would cause the donor to suffer grave harms, such as in the case of second kidney donation, they do not provide adequate reason to claim that for an instance of living organ donation to prove justified, a donor *must* receive benefits from donation that outweigh the harms she suffers.

This is self-evident where the threshold is justified via appeal to the dead donor rule and general moral injunctions against the deliberate/foreseen/negligent killing of innocents. It is also obvious where a threshold is imposed on the basis of concerns regarding the ethics of receiving. For, as a certain degree of sacrifice on the part of a gift giver is generally deemed acceptable, even in cases where gifts given and received are between strangers, there seems little reason to suggest that the same rules should not apply in the context of organ donation. Similarly, appeals to broader consequentialist considerations and respect for autonomy cannot ground such a strong threshold. On consequentialist approaches, after-all, whilst opportunities for donor benefit should be maximised where possible, benefit will not be required in all cases. For, if it is the case that net donor harm is *necessary* to secure greater benefits for others, and does not lead to wider negative consequences such as by risking the viability of transplantation programmes or reducing the number of willing living organ donors, such harms will be justifiable. In regards to autonomy too it can be noted that although an absence of donor benefit might sometimes act as a red flag, suggesting that a donor’s capacity to consent may be impaired, or that a lack of sufficient information has been provided regarding the likely harms and benefits of the procedure, this will not always be the case. For, provided a person is in possession of both the capacities and information required for an autonomous decision she may just as easily consent to being harmed as she may to being benefited.

So, given this, what might motivate the claim that donor benefit is required for justified living organ donation? For the main proponent of this view, Aaron Spital, whose work championing the donor benefit standard can be found in numerous medical and ethics journals (Spital 2004a, b, 2005a, b, 2006; Spital and Jacobs 2007; Spital and Taylor 2007, 2008) motivation is found in what he, and many others, consider to constitute the primary role-responsibility of the physician: to act always and firstly as unyielding advocate for his individual patient’s health and wellbeing (Levinsky 1984; Spital 2004a). This responsibility, Spital suggests, requires that any physician must—when considering whether or not to recommend a particular treatment or intervention for his patient—limit his concern solely to that of his patient’s welfare. The implications of this responsibility are clear. Just as a surgeon, in the process of amputating the gangrenous leg of his patient, is justified in operating by reference to the fact that the benefits of amputation for his patient outweigh the harms of surgery, the donor physician may only be justified in recommending his patient as a living organ donor when he is convinced that the expected benefits of donation for his patient (the donor) will outweigh the resulting harms (Spital 2004a, pp. 105–106).

This view of the physician’s primary obligation has its basis in two considerations. First of these is adherence to the well-known prima-facie principles of beneficence and non-maleficence. In combination, these require that when performing any medical intervention on a patient, a physician must be convinced that the intervention is more likely than not to provide benefits to his patient that outweigh the harms he or she might suffer (Spital 2004a, p. 105). Second, however, is the suggestion that just as the criminal lawyer “is obligated to use all ethical means to defend [his] client regardless of the cost of prolonged legal proceedings or even of the possibility that a guilty person may be acquitted through skillful advocacy” (Levinsky 1984, p. 1573) the physician must—for the sake of retaining both the individual patient’s and the public’s trust in medicine—exhibit a similar single-mindedness through acting solely as their patient’s advocate and thus ensuring that all that they do *to* their patient is done *for the sake of that patient*.

Spital therefore seems to suggest that although the practice of living organ donation may well be justified at a *societal* level by appeals to wider consequentialist considerations such as recipient benefit, such considerations should *not* serve as a guide for physicians when deciding whether to recommend their patient as an organ donor. For, the physician, unlike other members of society “is in the position of deciding not simply whether a subject’s choice is reasonable or morally justifiable but whether *he* [*whilst inhabiting his role as physician, not citizen*] is morally justified in helping the subject to accomplish it” (Elliott 1995, p. 95) He should decide whether or not to recommend his patient as a donor by considering only whether donation is likely to produce benefits for his patient “that are sufficient to offset the risks” (Spital 2004a, p. 106) of donation. Thus, Spital holds that a physician’s consideration of other factors, such as recipient benefit, poses - unless considered only as providing information regarding the extent to which a donor is liable to benefit from his donation—“a clear conflict of interest.” For, he suggests that to consider such factors asks “physicians to change their loyalty in a major and unacceptable way” as a physician may not fulfil his role “if, in trying to recommend a procedure [he] is asked to balance the risks for one patient against the benefits for another” (Spital 2004a, p. 106).

### What counts as a benefit on Spital’s view?

With Spital’s position laid out above it becomes prudent to explore the *kinds* of benefit to which he is referring when he states that donor benefit is necessary. The benefits the living organ donor may accrue, as a result of their donation, are not likely to be ‘physical’. The living organ donor—regardless of the organ/s donated, and the severity and longevity of the harms associated with their donation—will always leave the hospital physically diminished and at a risk of developing complications because of their donation. As Den Hartogh succinctly puts it, “the explantation of living organs harms the donor; it is a medical intervention that does not turn a patient into a healthy person, but rather turns a healthy person into a patient” (Den Hartogh 2013, p. 45).

Instead, what Spital has in mind are ‘psychological and emotional benefits’, including an improved quality of life and lasting increases in self-esteem due to the knowledge that they have helped to save a life and “seeing a loved one resurrected and then having that cherished person available for sharing the joys of life” (Spital 2004a, p. 107). These, Spital claims, are “at least as important as physical ones” (Spital 2004a, p. 107) for the overall wellbeing of individuals. Spital does recognise, however, that the physician is not ideally placed to determine the extent to which a particular donor is liable to receive psychological and emotional benefits. Such benefits are “subjective”, with their weight and intensity depending “heavily on individual values and life plans, the details of which are only available to the volunteer” (Spital 2004a, p. 108). Thus, Spital suggests that after providing adequate information regarding donor risks (both physical and psychological^1^), and the likelihood of success for the recipient, and ensuring sufficient understanding on the part of the donor, a donor’s physician must rely heavily on the testimony of the living donor when making a risk–benefit calculation. For, he notes, “it is the potential donor who can best assess how likely she is to benefit and whether anticipated nonmedical benefits are worth the risks, because only she knows and understands what is most important to her” (Spital 2004a, p. 108).

That living organ donors may, and often will, receive sufficient psychological and emotional benefits that the harms and risks of donation may be justified per the donor benefit standard, and that he or she may well be harmed more by a physician’s decision to refuse donation than one to permit donation, is supported by the studies concerning the outcomes of living organ donors. These show that few living donors regret their donation, even where recipient outcomes are unfavourable, and most report increases in overall quality of life after their donation: believing that they have gained more psychologically than they have lost physically (Reese et al. 2015; Weidebusch et al. 2009; Clemens et al. 2006). This, however, is unsurprising. Studies regarding donor motivation show that, despite the widespread belief that the primary motivators for organ donation are based in solidarity and an altruistic concern for the wellbeing of others, this is not the case. Many organ donors are not pure altruists: willing to sacrifice their own interests for the sake of another with no expectation of, or desire for, benefit. Instead, they hold mixed and complicated motivations for donation.

Some of these motives may, of course, be *purely* other-regarding—and thus would, where attained, be unable to justify living donation on Spital’s view—such as a desire to save or improve the life of a loved one (or stranger) for the *recipient’s sake alone*. Many others, however, *are* purely self-regarding (aimed at securing benefits and avoiding or mitigating harms for themselves), or self-interestedly other-regarding (aimed at securing benefits for others because the donor’s welfare interests are intimately bound up with the wellbeing of their intended recipient). Some benefits sought are dependent on the outcome of the transplant for the recipient, especially in cases of the directed donation of organs to emotionally related recipients where the donor has direct and vicarious welfare interests in their intended recipient’s survival (Wilkinson 2011, pp. 124–125). These include, but are not limited to, a desire to avoid the grief and suffering that results from the death of a close friend or relative, to see their loved one in good health and enjoying life once more, to improve family life and relationships that may have been strained by a prolonged period of illness, to be able to enjoy activities such as hobbies and travel with the recipient, to relinquish the role of caregiver, and even to improve one’s financial position once the recipient returns to work. Other benefits however, are not dependent on recipient outcome but upon the act of donation. Donors for example, may seek and find comfort in the knowledge that they have done all they can to save a loved one even if the transplant is unsuccessful; they may wish for praise from others or to be perceived as ‘good’ or ‘generous’; and may also use the act of donation to improve their own self-esteem, atone for past wrongdoing or, indeed, as a means of paying their own good fortune forward (Challenor and Watts 2014, p. 399).

### Implications for donors and recipients

Would the donor benefit standard, if implemented, lead to a reduction in the number of organs available for transplantation? Glannon and Ross suggest, in a short piece criticising this and other aspects of Spital’s work that a reduction in the number of organs made available for transplantation would be the likely result (Glannon and Ross 2005, p. 193). This is, of course, possible. However, as can be seen from the section above this impact would likely be small as many organ donors (both related and ‘stranger donors’) do *seek and receive* benefits that they deem (and thus the physician adhering to the donor benefit standard would likely deem) sufficient to outweigh the harms they suffer.

Acceptance of the donor benefit standard in the context of living organ donation may, however, also have consequences for other forms of donation. For, although practices such as blood donation would likely be unaffected by implementation of the donor benefit standard as it can be claimed in such cases that the risks are so small that they can easily be justified by appeal to the psychological benefits associated with altruistic behaviour and through the maintenance of a system of blood provision that is to everyone’s benefit, this may not be the case in the context of practices such as ova donation for the purposes of reproduction and research. This is so as while the long-term risks of ova donation are unknown given the relative infancy of the procedure, studies have shown that it has the potential to impose significant harms on donors. These include discomfort and pain from daily hormone injections, rapid weight gain and respiratory difficulty, damage to other organs such as the bladder, bowel and uterus as a result of the egg retrieval procedure, and decreased fertility, infertility, ovarian hyperstimulation syndrome, life threatening haemorrhage, thromboembolism and an increased risk for ovarian, breast and colon cancer (Baylis 2013, p. 533). Thus, although in many cases—such as where donation occurs between friends and family members—the risks of ova donation may be outweighed by the benefits associated with seeing a loved one fulfil a deeply held desire to parent, this will not always be the case. In cases where donation occurs between strangers:

[T]the harm–benefit ratio is not obviously favourable, in part because the benefits and harms devolve on to different people. The [egg receiver] has the potential benefit of having a child. The egg provider bears the potential harms of hormonal stimulation and egg retrieval; her only potential benefits are emotional (a good feeling from an act of altruism) and possibly financial (if payment is involved). Whether these potential benefits compensate for the potential harms is a contested matter. (Baylis 2013, p. 533)

That the donor benefit standard may well reduce the number of organs available for transplantation (and have implications for practices such as ova donation) when compared to existing and less restrictive standards is, of course, regrettable. It does not, however, *necessarily* provide sufficient reason to abandon it. For, as Spital correctly notes in a response to Glannon and Ross—*if* the donor benefit standard overcomes the shortcomings associated with other standards for justified living organ donation, and does not fall prey to serious criticisms on other grounds - that the donor benefit standard will reduce the number of organs available for transplantation would only prove a decisive reason to abandon it *if* it can be shown that there is “*another* ethically acceptable method for determining the suitability of volunteers that [yields] more donors” (Spital 2005a, pp. 197–198). Unfortunately for Spital however, it is far from apparent that the donor benefit standard does provide advantages over existing and proposed methods that yield more donors, or that it does not fall prey to other serious criticisms on different grounds. With this in mind, within the following sections I demonstrate through an analysis of the advantages Spital claims for his position that these are either non-existent or flawed and thus provide scant reason to adopt the donor benefit standard.

## On the purported advantages of the donor benefit standard

In his papers Spital suggests that if accepted and implemented the donor benefit standard would provide a number of clear advantages when compared to currently existing criteria for justified living organ donation. These can be summarised as follows:

1. The donor benefit standard is *the only standard* for justified living organ donation that is “consistent with the clear benefit standard used to determine the acceptability of incompetents as donors” (Spital 2004a, p. 106).
2. The donor benefit standard “accommodates and considers individual values” (Spital 2004a, p. 106).
3. The donor benefit standard “avoids conflicts of interests that occur under the donor risk-recipient benefit balancing plan… and directs physician loyalty to where it belongs—solely with her patient” (Spital 2004a, p. 106).

### ‘Incompetent donation’ and the value of consistency

Although donor benefit has not, until relatively recently, been proposed as a necessary requirement for justified living organ donation, it is widely accepted that donor benefit is a necessary requirement of justified organ retrieval in the rare cases where those who lack the capacity to consent—such as the very young and the intellectually disabled—are proposed as organ and tissue ‘donors’.^2^

In such cases, although some maintain that such persons should never be used as sources of organs for transplantation given their vulnerability to exploitation and abuse (see, for example: Webb and Fortune 2006; Cheyette 2000), it is more commonly suggested that an outright ban on this practice would serve neither the interests of potential organ recipients nor those of the proposed ‘donors’ themselves (Munson 2002; Wilkinson 2011). For, provided physical, psychological and emotional harms and benefits are taken into account those who lack the capacity to consent may, just like competent donors, suffer greater harms from a decision to forbid organ retrieval than one to permit it.

Thus, it is conventionally held that, in these rare cases, organ retrieval may be justified in the absence of capacity to consent where it can be shown: firstly, that there are no alternative competent sources of donor organs; and secondly, that despite the harms of retrieval, the donor is likely to receive sufficient psychological and emotional benefits that they outweigh the harms inflicted. When, how, and indeed *whether* such a determination can be made is, of course, questionable given the difficulties associated with donning the mental mantle of another (and especially that of another whose psychology is importantly different from one’s own). However, difficulties aside, in cases where it *can* be shown that retrieval is in the interests of the ‘donor’—and provided adequately and stringently policed safeguards are implemented to protect against exploitation and abuse—retrieval will be to the advantage of both ‘donor’ and recipient.

This view has not only been advanced in the academic literature but has also been accepted and implemented by the courts in a number of jurisdictions, such that it has played a *key role* in legal cases where persons lacking capacity have been proposed as organ and tissue ‘donors’ since the 1950s. In the US, for example, in the case of *Masden v Harrison* kidney donation from a 19-year old (who was under the age of majority at the time) to his identical twin brother was permitted by a Massachusetts court as a result of testimony from a psychiatrist that “grave emotional impact may be visited upon [the donor] if the defendants refuse to perform this operation”, and thus that the operation was necessary for the “continued good health and future wellbeing of [the donor]” (Curran 1959, p. 893).^3^ Similarly, in the 1972 case of *Hart v Brown*, a Connecticut court permitted kidney donation from a 7-year old to her twin sister as a result of psychological testimony which suggested that the procedure would benefit the donor due to her close emotional relationship with her sister and in the form of a happier family life (Hart v Brown 1972)

Donation from incompetent *adults* has also been justified in the US on the basis of such considerations, such as in the 1969 case of *Strunk v Strunk* where the donation of a kidney from a 27-year old with severe cognitive problems to his brother was permitted by a Kentucky court. In this case, it was held that because the donor was dependent on his brother both “emotionally and psychologically… his wellbeing would be jeopardised more severely by the loss of his brother than by the removal of a kidney” (Strunk v Strunk 1969). In the UK too, although young children and the intellectually disabled have never been used as organ donors (Webb and Fortune 2006), cases have been recorded where individuals lacking capacity have been used as bone marrow donors by reference to testimony that donation was in their best interests. In the 1997 case of *Re Y*, for example, the courts permitted bone marrow retrieval from Y, a severely mentally and physically disabled adult to save the life of her sister after expert testimony that whilst Y and her sister did not share close emotional ties, Y was extremely close to her mother whose health—and thus, ability to visit Y—would be negatively impacted by Y’s sister’s death (Re Y 1996).^4^

With such cases in mind, Spital suggests that *because* a ‘clear benefit standard’ is often used to determine the appropriateness of donation in cases from those who lack capacity it would be inconsistent to claim that the same rules and protections should not apply in the case of competent volunteers. This kind of argument is promising as, should it be the case that to impose different standards on the retrieval of organs from competent and incompetent donors is inconsistent, this would provide some reason to accept and thus implement the donor benefit standard in the case of competent volunteers. After all, despite the derisory opinions of authors such as Huxley who held that consistency is “contrary to nature, contrary to life” (Huxley 1931, p. 125) and Wilde who suggested it to be “the last refuge of the unimaginative” (Wilde 1885), it is generally held, for reasons of both fairness and coherence that like cases should be treated alike and thus that consistency *is* something to be sought in the principles and laws that govern our behaviours.

Despite this, the suggestion that the extension of the donor benefit standard from cases of incompetent to competent donors should be seen as a mark of consistency is mistaken. It seems to ignore the fact that, where those who lack the capacity to consent are proposed as ‘donors’, philosophers and the courts are only concerned with the overall balance of donor benefit to burden *because they are concerned to act in the donor’s interests were she able to articulate them*. In other words, a concern with donor benefit is subordinate to a concern to act in the candidate ‘donor’s’ interests. Benefit is desired, but it is desired as a means to an end, rather than for its own sake. Given this, it seems that if Spital and other proponents of the donor benefit standard are *truly* concerned with ensuring consistency in the treatment of competent and incompetent living donors, a focus on ensuring donation is in the interests of candidate donors, rather than ensuring a positive balance of harm to benefit, is in order.

### Welfare interests and the other regarding concerns of competent donors

Very young children and severely intellectually disabled persons lack a number of interests commonly ascribed to most adults for reasons relating both to their levels of cognitive development (which may preclude their possession of certain, more complex, interests) and communication skills (which makes it difficult to determine what is truly in their interests). As such, it is generally considered safer to attribute to them *only* egoistic interests in their own wellbeing when it comes to decisions with the potential to significantly affect their welfare. We know, for example that the very young and persons with severe intellectual disabilities will, like all sentient creatures, possess interests in avoiding pain and experiencing pleasure. We do not know, however, if such individuals possess interests in restoring the health of a sick relative except when doing so is likely to serve their welfare interests, such as through maintaining a close and emotionally beneficial relationship or avoiding the pain and confusion that may be caused by their death. A focus, therefore, on the extent to which an incompetent donor will be harmed or benefited seems appropriate in such cases.

Yet, whilst it is prudent to ascribe to young children and persons with severe intellectual disabilities *only* self-regarding interests in the context of living organ donation, this will not be the case for most adults. For, their interests do not, except perhaps in cases of those with severe antisocial personality disorders, reduce merely to those of direct and indirect/vicarious interests in their own wellbeing. They also, and importantly, tend to include interests in the interests of others. This is because mature human beings are not, despite the picture painted by many, essentially or ideally independent creatures who are disengaged from others and characterised by self-interest. They are instead socially embedded entities who are “intimately related to other people, groups, institutions and histories” and are thus “motivated by interests and reasons that can only be fully defined with reference to other people and things” (Christman 2004, p. 144).

That such is the case is well-supported in the philosophical literature. Dworkin, for example, suggests that alongside the ‘experiential interests’ persons possess in having desirable ‘felt’ experiences such as comfort and enjoyment and avoiding undesired experiences such as pain, they also possess ‘critical interests’ in having what they value or care about becoming a reality. These may include but are not limited to parental or spousal interests in their children’s or partner’s success and wellbeing, a desire to ameliorate suffering in the world, or to preserve beauty in the world (Dworkin 1993, pp. 201–208). Similarly, Feinberg suggests that persons possess, in addition to interests in their own welfare which may be seen as purely self-regarding, what he terms ‘ulterior interests’. These, he suggests, are a person’s more “ultimate goals and aspirations” (Feinberg 1987, p. 37) and include both “want regarding interests” (Barry 1965, p. 183) in personal fulfilment such as through “producing good novels or works of art… [or] achieving high political office” (Feinberg 1987, p. 37), but also, and importantly, what may be termed “ideal regarding interests” (Barry 1965, p. 183) in such things as “advancing a social cause, ameliorating human suffering, achieving spiritual grace” (Feinberg 1987, p. 37).

With this in mind, we can note that just as a competent person (A) can possess an interest in the wellbeing of another (B) for purely self-regarding reasons (*because* what promotes B’s welfare interests also promotes A’s welfare interests directly or indirectly) he may also have interests in B’s wellbeing for reasons that fail to regard his own welfare, but regard instead the emotional, social, and moral relationships in which he stands to other human beings. These may include both:

1. *Loving interests* A has an interest in promoting/securing the wellbeing of B *because*, as a result of the emotional and social relationship in which he stands to B, “he desires B’s good *not simply* as a means to the promotion of the other ulterior aims that are components of his own good, but quite sincerely as an end in itself” (Feinberg 1987, p. 71).
2. *Ideal-regarding interests* A has an interest in B’s interests *because* as a result of his personal values and moral commitments (i.e. a desire to increase levels of happiness in the world or a desire to decrease inequality) B’s interests are in his personal interest.

Extended to the case of organ donation, A may have an interest in donating an organ to B both *because* A will be either directly or vicariously harmed should he be barred from donating but also, and importantly, because as a result of the relation in which he stands to B (mother, brother, wife, friend, fellow human being etc.) A has *purely other regarding interests* in B’s wellbeing. That is, an individual may possess an interest in donating an organ to another not simply as a means to securing his own future wellbeing but also because as a result of both the moral and emotional relationships that obtain between individuals, he possesses an interest in donating an organ for the recipient’s own sake.

Of course, whilst the distinction between self-regarding and other/ideal-regarding interests might be clear in theory this will not always be the case in practice. Loving or purely other-regarding interests are “commonly intertwined with and reinforced by… self-regarding interests” as “most of the things we desire for their own sakes we also desire as a means to other things” (Feinberg 1987, p. 72). A husband or father, after all, may be motivated to donate an organ to his daughter or wife primarily because her declining health violates the other-regarding interests in her welfare he possesses as a result of his love for her, but also because it is instrumentally damaging to various of his self-regarding interests: constituting a drain on economic resources, a burden on his time and energy, a strain on his emotional stability etc. Similarly, a woman who donates an organ, such as a kidney, or, indeed, a uterus, to a stranger may well be motivated by a commitment to alleviate the suffering of another and at the same time, by numerous self-regarding reasons: because seeing suffering in the world causes *her* emotional distress and/or because she desires to be viewed by others as ‘good’ or ‘virtuous’ etc.

Given this, it seems that the extension of the donor benefit standard from cases of ‘donation’ involving minors and those with intellectual disabilities to cases of donation from autonomous adults is inconsistent with the motivation which underlies its acceptance in the former case. For, whilst when applied to cases of ‘donation’ involving minors and the intellectually disabled, the standard will, more often than not, result in outcomes that can be said to be in the interests of the ‘donor’, this will not be so when it is applied to competent volunteers. As shown above, the donor benefit standard’s sole focus on welfare interests discounts a vast swathe of human interests which may arise from the emotional bonds characterising normal and loving human relationships as well as a number of less common interests and values, such as that of selfless concern for the interests of others motivated by altruism and other strongly held moral commitments and ideals which may be frustrated should the candidate donor’s offer be refused.

Similarly, that the donor benefit standard discounts purely other-regarding interests and values such as altruism and solidarity casts serious doubt on Spital’s claim that the donor benefit standard “accommodates and considers individual values” (Spital 2004a, p. 106). For, while the donor benefit standard directs welcome attention to the *interests of living donors*, many of whom do seek benefits from donation, it seems odd and more than a little paternalistic to suggest that in cases where candidate donors are primarily motivated by prosocial and other-regarding values, donation may be justified *only* where a physician is convinced that a donor will attain *undesired* (albeit not necessarily unwelcome) benefits.

Importantly then, in order to prove truly consistent with existing moral standards for the treatment of incompetent donors *and* in order truly to accommodate and consider the individual values of mature human beings, proponents of the donor benefit standard (as laid out by Spital) *should* adjust their focus from benefits to that of ‘interests’ more broadly construed. This, however, would require that transplant physicians be *permitted* to retrieve organs from living donors in cases both where they are convinced that donation serves the welfare interests of donors (providing *them* with emotional, psychological and social benefits that outweigh the harms they suffer) and/or where donation results in net donor harm but forwards the other-regarding interests of donors.

#### On the primary role-responsibility of the physician

As seen in the preceding subsection, the donor benefit standard is neither consistent with existing standards for the retrieval of organs from persons lacking capacity to consent to their donation nor able to truly accommodate and consider the individual values of competent organ donors. Provided, however, its proponents are willing to expand their account such that donation is justified where it can be shown to serve the *interests* of candidate donors - where interests are more broadly construed and include both self-regarding and other regarding interests—such criticisms *can be* avoided. Despite this, however, it is suggested in this section that an additional criticism of the donor benefit standard may not be so easily solved by emendation.

This criticism attaches to the claim at the heart of the donor benefit standard: the suggestion that for a physician adequately to *fulfil her role* she must believe that donation provides benefits for her patient that offset the harms of donation and thus that living organ donation absent this requirement poses a *clear and problematic conflict of interest for the physician*.^5^

Now, Spital *seems* in good company when he makes this claim. The suggestion that a doctor’s role should primarily be that of his patient’s advocate and thus that he should always endeavour to act in their best interests has historically played, and continues to play, a central role in medical ethics. It is, through “images of the physician at the bedside, dedicated to his present patient… consecrated in art and literature” (Wendler 2010, p. 66) and is enshrined still in virtually all national and international guidelines and legislation governing the practice of medicine. In the UK, for example, the General Medical Council’s guidelines for ‘Good Medical Practice’ instruct the physician to “make the care of their patient their first concern.” (General Medical Council 2013, p. 6) The Canadian Medical Association requires too that the physician must “consider first the wellbeing of the patient” and “take all reasonable steps to prevent harm to patients” (Canadian Medical Association 2004, pp. 1–2). Similarly, the American Medical Association requires that “A physician shall, while caring for a patient regard responsibility to the patient as paramount” (American Medical Association 2016, p. 1), the American College of Physicians stipulates that the “physician’s primary commitment must always be to the patient’s welfare and best interests” (Snyder 2012, p. 75) and the World Medical Association holds that “A physician shall owe his/her patients complete loyalty” and “act in the patient’s best interests when providing medical care” (World Medical Association 2006).

Yet, although there is widespread endorsement of this view, close examination of both medical practice and medical guidelines shows that there is also widespread endorsement of the view that the physician’s obligation to act in the best interests of her patients admits of numerous exceptions and must be balanced against other considerations.

Indeed, Wendler notes that there are, in fact, 27 exceptions to this rule which are generally considered appropriate (Wendler 2010, p. 66). Many of these, he suggests, can be traced to the simple fact that physicians are often patient-rich but time-poor and thus “cannot always promote the present patient’s interests without undermining the medical care of their other patients.” (Wendler 2010, p. 66) The physician, after all, is not generally criticised when he leaves one patient’s bedside to respond to code, postpones the care of one patient to administer care to another who is in more immediate need, or is forced to divide his time between the care of his own patients and those of a sick colleague. Some exceptions to this rule, however, are based in other considerations. These include, but are not limited to: respect for patient autonomy, such as in cases where less effective alternatives to blood transfusions are used in surgery in order to respect the religious beliefs of Jehovah’s Witnesses; respect for physician autonomy such as where physicians are permitted, on the basis of religious and other beliefs, to refuse to perform certain medical interventions such as abortions and instead refer those who seek them to other physicians willing to perform such procedures; public health and other societal interests such as where a physician may refuse antibiotics to patients in order to avoid resistance or quarantine and report infectious diseases to public health officials to protect the wider public, and provide less effective and less expensive medication in order to preserve medical resources for others; for the purposes of physician training; *and* the wellbeing of physicians such as by ensuring their working hours are manageable, and allowing them such luxuries as holidays and retirement even though to do so may sometimes compromise the care of individual patients (Wendler 2010, p. 67).

The physician’s role is far more complex than Spital assumes, and the obligation of the physician to act as patient advocate should, in fact, be seen to constitute a *pro tanto* obligation rather than an absolute one. It is important but must also be balanced alongside competing considerations. In the context of living organ donation many of these considerations may not apply. Yet some, such as respect for the autonomy and religious beliefs of candidate donors, as well as a wider concern with the health and welfare of other members of society do seem relevant and thus cast doubt on the suggestion that in the context of living organ donation a physician may *never be justified* in retrieving organs unless he is convinced that to do so will be to the benefit of his individual patient. Given this, it seems inconsistent to suggest that a physician may not appeal to similar considerations to justify retrieval in cases where a competent donor is unlikely to benefit on balance from her donation but is also unlikely to suffer such significant and enduring harms from retrieval that his capacity to consent may be questioned.

## Conclusion

This paper has examined the claim that commonly accepted criteria for justified living organ donation from competent volunteers—which encompass the requirements that living donors provide their valid consent to donation, that living donation produces an overall positive balance of benefit to burden for those holding a stake in the donation which cannot be achieved in a less harmful manner, and that living donors are not liable to suffer grave harms as a result of their donation—are not sufficient to offer a general account of justified living organ donation. As noted in the introduction, this claim may have several bases, but this paper has been concerned with just one: the suggestion that in order to prove justified an instance of living donation should be reasonably expected to provide benefits for the donor that outweigh the harms that they may suffer, a position I termed ‘the donor benefit standard’.

After exploring the possible motivations for the imposition of weaker versions of the donor harm threshold, an argument provided by Aaron Spital in numerous papers was explained. This position suggests that in order to: ensure consistency between our treatment of incompetent and competent donors; truly respect and consider the individual values of candidate donors; and avoid conflicts of interests for physicians whose primary role responsibility is that of advocate for her patients, any standard (or set of standards for justified living organ donation) which does not require that the living organ donor receives benefits from their donation outweighing the harms they suffer is insufficient.

However, this paper claims that although the donor benefit standard directs welcome attention to the interests of living organ donors whose motives for donation are not generally wholly other-regarding, crystallizing the importance of ensuring clear and candid discussions among donors and physicians regarding their motives, there is scant reason to think that a blanket imposition of the donor benefit standard would improve on currently existing standards for justified living organ donation. For, analysis shows both that the benefits commonly claimed by proponents of the donor benefit standard are illusory or flawed and that its imposition would pose significant threats to the autonomy of candidate donors and the lives of those who may benefit from organ transplants. Given this, it is concluded that wherever the donor harm threshold is placed (if at all) in living organ donation, it should not be so strong as to require that donors receive sufficient benefits that they outweigh the harms of donation.

## Footnotes

1 While the physical perils of living organ donation are well documented, living organ donation also poses a small risk of the donor’s experiencing significant psychological harms such as risk of suicide and/or other psychological sequelae should he or she suffer severe complications or where the transplant fails (for more information see, for example Jowsey and Schneekloth 2008; Erim et al. 2006).

2 The term donor placed in scare quotes here (although not throughout the rest of the section for stylistic reasons) in recognition of the fact that donation generally implies a willing transfer on the part of the donor, which, of course, is notably lacking in cases where those who lack the capacity to consent are proposed as sources of organs.

3 N.B. The case of *Masden v Harrison* is unreported and Curran’s paper is the earliest containing excerpts from the slip opinion of the court.

4 The extent to which Y served to benefit because of her donation in this case has been the subject of significant debate amongst medical lawyers. (For commentary on this case see, for example Feenan 1997).

5 It should also be noted that just as Spital problematically equates the Best Interests Standard with his own Donor Benefit Standard when used to determine the moral acceptability of organ donation in the case of incompetent donors, he seems to do the same here. For, *even if* we accept that a physician’s primary obligation *is* to act as advocate for his individual patients and that to do otherwise poses a clear conflict of interest there is little reason to assume that such advocacy should extend *only* to forwarding the direct and vicarious welfare interests of living organ donors.
